# Carrot Discard as a Promising Feedstock to Produce 2,3-Butanediol by Fermentation with *P. polymyxa* DSM 365

**DOI:** 10.3390/bioengineering10080937

**Published:** 2023-08-07

**Authors:** Juan Carlos López-Linares, Adrián Mateo Martínez, Mónica Coca, Susana Lucas, María Teresa García-Cubero

**Affiliations:** 1 Department of Chemical Engineering and Environmental Technology, School of Industrial Engineering, University of Valladolid, Dr. Mergelina, s/n, 47011 Valladolid, Spain; juancarlos.lopez.linares@uva.es (J.C.L.-L.); adrian.mateo.martinez@alumnos.uva.es (A.M.M.); monica.coca@uva.es (M.C.); susana.lucas.yague@uva.es (S.L.); 2 Institute of Sustainable Processes, University of Valladolid, Dr. Mergelina, s/n, 47011 Valladolid, Spain

**Keywords:** carrot discard, enzymatic hydrolysis, semi-defined media, 2,3-butanediol, *Paenibacillus polymyxa*

## Abstract

The valorization of fruit and vegetable residues (such as carrot discard) and their microbial conversion into 2,3-butanediol (BDO) can be considered as a very interesting way to reduce food waste and sustainably originate high value-added products. This work analyzes the valorization of carrot discard as feedstock for 2,3-butanediol (BDO) production by *Paenibacillus polymyxa* DSM 365. The influences of stirring and the presence of tryptone (nitrogen source) are studied. Furthermore, in order to evaluate the influence of the pre-culture medium (nitrogen source, nutrients, and pH) and the substrate, fermentation assays in simple and mixture semi-defined media (glucose, fructose, and/or galactose) were also carried out. As a result, 18.8 g/L BDO, with a BDO yield of 0.43 g/g (86% of its theoretical value), could be obtained from carrot discard enzymatic hydrolysate at 100 rpm, no tryptone, and pre-culture Häßler medium. No hydrothermal pre-treatment was necessary for BDO production from carrot discard, which increases the profitability of the process. Therefore, 18.8 g BDO, as well as 2.5 g ethanol and 2.1 g acetoin by-products, could be obtained from 100 g of carrot discard (dry matter).

## 1. Introduction

2,3-butanediol (BDO) is regarded as an important industrial platform, bulk, and fine chemical, as well as a valuable commercial chemical [1,2]. Among its main properties are the fact that it is colorless, odorless, transparent, and hygroscopic, as well as having a high solubility in water (500 g/L (20 °C), alcohols, ketones, and ethers, with a good biodegradability [3]. Therefore, BDO has found applications in the polymer, cosmetics, fuel, and painting industries [2,4]. It can be used as an intermediate for the production of solvents and high value-added products, such as methyl ethyl ketone (employed in the coating, lubricant, and adhesive industries), gamma-butyrolactone (a flavoring and cleaning solvent), 1,3-butadiene (used as synthetic rubber), polyurethanes, and acetoin and diacetyl (flavor enhancers) [3,5]. Moreover, it can be used as anti-freeze because of its low freezing point (−60 °C, 1 atm), as an “octane booster” for gasoline, as a liquid fuel (high heating value of 27,198 J/g), and as an ink additive, food additive, and fumigant [1,3].

Nowadays, BDO is generated in industries by the cracking of petroleum-derived hydrocarbons (butane and 2-butene). Nevertheless, due to the recent unsteadiness in the price of petroleum, there has been increased interest in the potential of BDO microbial production from cheap agro-industrial residues, with a potential also for large-scale production, and this is expected to considerably reduce its overall production cost [3]. In this context, according to Maina et al. [5], it is expected that the BDO global market would increase to USD 220 million by 2027, with a growing compound annual growth rate (CAGR) of 3% from 2019 to 2027.

BDO can be produced via fermentation together with acetoin that is considered one of the most important sugar-derived platform chemicals. Acetoin can be found in fruit, vegetable flours, butter, cocoa, vinegar, and wine, among others. Moreover, it is widely used in the food (as a taste improver) and cosmetic (as a fragrance agent) industries, as a precursor to synthesize chelating agents (a platform compound), as well as in the microbiology, botany, pharmaceutical, and agriculture sectors [5,6].

Different bacteria, such as *Klebisella*, *Bacilus*, *Enterobacter*, *Ralstonia*, *Paenibacillus,* and *Serratia marcescens*, or even *Saccharomyces cerevisiae* mutant yeasts (i.e., YG01_SDBN and YPH499/pol3δ/BD_392), among others, are able to produce BDO [3,7]. Among them, *Paenibacillus polymyxa* is highlighted, since it is a non-pathogenic (class 1) strain with a high potential for BDO production. It could, therefore, be suitable for industrial-scale fermentation, as there is no biological safety level to consider [8]. These bacteria synthesize BDO through a complex metabolic pathway, where the substrate (mainly glucose) is first converted into pyruvate, to later become BDO through successive pathways, with α-acetolactate synthase, α-acetolactate decarboxylase, and acetoin reductase (2,3-butanediol dehydrogenase) enzymes [9].

These microorganisms are able to use a wide range of six- and five-carbon sugars as carbon sources, such as glucose, fructose, xylose, ribose, and arabinose, among others [10]. BDO production from different lignocellulosic residues, such as corn stover [11], sweet sorghum stalk [12], sugar beet pulp [13], soybean hull [14], sugarcane bagasse [15], oil palm frond [16], apple pomace [17], rice straw [18], and fruit and vegetable residues [19] has been reported in the literature. Most of these residues present complex structures, cellulose, and hemicellulose, which require a pre-treatment step in order to obtain the simple sugars [15]. In addition, *Paenibacillus* species and specifically *P. polymyxa* are able to ferment carbohydrate polymers (such as xylan, inulin, and starch), secreting xylanase, inulase, and α-amylase; while simultaneously converting those polymers to monosugars [8].

The use of agro-industrial residues, such as fruit and vegetable residues, is essential in a circular economy to make this sector much more efficient and sustainable [15] since they can be used to produce high value-added products (i.e., bioactive compounds) [20], fine chemicals, platform chemicals, and/or biofuels [21,22]. Carrot, with an annual production in 2020 of 36 Mt [23] and 0.4 Mt [24] worldwide and in Spain, respectively, presents an interesting composition of both free sugars and structural carbohydrates [25]. Moreover, between 25% and 30% of carrot production is discarded due to physical defects and non-conformity for the market [23], reducing the 2,3-BDO production cost

On the other hand, although the generation of bioethanol using carrot discard (CD) has been reported in the literature [26], to the best of our knowledge, this is the first work on BDO production by fermentation.

The objective of this study is to evaluate the production of BDO from enzymatic hydrolysate of carrot discard by *P. polymyxa*. First, the preference for sugar uptake by the microorganism was evaluated for different growth media. Then, the influence of stirring parameters and the presence of tryptone (as the organic nitrogen source) was also analyzed in terms of BDO concentration and productivity.

## 2. Materials and Methods

### 2.1. Raw Material

CD, which was kindly supplied by a vegetable company (Horcaol Cooperative Society, Olmedo, Valladolid, Spain), was milled to a particle size of 1–3 mm using a household grinder and stored at 4 °C before being used in enzymatic hydrolysis and BDO fermentation assays. The composition was (% *w*/*w* dry matter): galacturonic acid, 11.2 ± 0.2; cellulose, 11.2 ± 0.1; hemicellulose, 5.5 ± 0.3 (galactose + fructose, 4.2 ± 0.2; arabinose, 2.0 ± 0.2); acid-insoluble lignin (AIL), 0.3 ± 0.0; acid-soluble lignin (ASL), 1.6 ± 0.0; extractives, 58.8 ± 0.4 (water extractives, 42.6 ± 0.3); (galacturonic acid in water extractives, 1.2 ± 0.0; glucose in water extractives, 15.3 ± 1.9; galactose + fructose in water extractives, 12.6 ± 1.3; arabinose in water extractives, 0.7 ± 0.3; ethanol extractives, 16.1 ± 0.4); ash, 7.5 ± 0.4; and acetyl groups, 0.6 ± 0.0.

### 2.2. Enzymatic Hydrolysate of Carrot Discard

In order to obtain the enzymatic hydrolysate of CD, an enzymatic hydrolysis process with CD as substrate (10% *w*/*v* loading: 25 g substrate and 250 mL enzymatic solution) was carried out in 1000 mL Erlenmeyer flasks at 50 °C, atmospheric pressure, 150 rpm, 24 h, and pH 4.8, employing an orbital shaker (Optic Ivymen Systems, Comecta, Barcelona, Spain). The solvent used was water, the pH being set to 4.8 with potassium hydroxide (KOH) 10 M at the beginning and throughout the process. A mixture of Cellic CTec2 and Viscozyme L enzymes (enzymatic activity of 90 and 54.5 filter paper units (FPU)/mL, respectively), kindly donated by Novozymes A/S (Bagsvaerd, Denmark), was used at an enzyme load of 10 FPU/g substrate for both enzymes. The conditions of enzymatic hydrolysis were selected on the basis of previous results. After completing the enzymatic hydrolysis, the enzymatic hydrolysate obtained was vacuum filtrated, its sugar content was measured, and it was finally used as the fermentation medium in BDO fermentation production.

### 2.3. Microorganism and Inoculum

The microorganism employed in the BDO fermentation was *Paenibacillus polymyxa* DSM 365, from the German Collection of Microorganisms (DSMZ, Braunschweig, Germany). The strain was reactivated by inoculating the lyophilized cells into DSMZ liquid medium and growing them overnight (12 h) at 30 °C in an orbital shaker (Optic Ivymen Systems, Comecta, Spain). The composition of the DSMZ liquid medium was (g/L): peptone, 5; meat extract, 3; and MnSO_4_.H_2_O, 0.01, at pH 7. The strain was stored as glycerol stock (40% (*v*/*v*) sterile glycerol) at –80 °C until further use.

The inoculum was grown using two different pre-culture media chosen from the literature after performing a previous literature review: Häßler (H) [27] and Okonkwo (O) [28]. The composition of the medium H [26] was: 20 g/L glucose, 10 g/L yeast extract, 0.2 g/L MgSO_4_, 3 g/L (NH_4_)_2_SO_4_, 100 mM potassium phosphate buffer (pH 6), and 3 mL trace elements. However, the medium O [27] was composed of: 20 g/L glucose, 5 g/L yeast extract, 5 g/L tryptone, 0.2 g/L MgSO_4_, 3 g/L (NH_4_)_2_SO_4_, 0.9 mL phosphate buffer (pH 6.5) (formed by (g/L): KH_2_PO_4_, 3.5; K_2_HPO_4_, 2.75), and 0.09 mL trace elements. In both media, the trace element solution was prepared according to Häßler et al. [27].

Regarding the medium H, the culture was grown in 250 mL Erlenmeyer flasks, containing 100 mL of medium; while for the medium O, the inoculum was grown in 100 mL Erlenmeyer flasks, containing 30 mL of medium. Both media H and O were sterilized at 121 °C for 15 min; while the potassium phosphate buffers (pH 6 and 6.5) and trace element solutions were prepared separately and sterilized by filtration using 0.2 μm cellulose nitrate filters (Sartorius 254 stedim Biotech, Göttingen, Germany). For both media, 1 mL of *P. polymyxa* glycerol stock was inoculated. The cells were grown in a rotary shaker at 37 °C and 200 rpm for 24 h (medium H) and 10–12 h (medium O). In the case of the medium O, at 10–12 h of growth (with an optical density at 600 nm (OD_600nm_) of about 1.0–1.2), 10 mL of actively growing cells was re-inoculated in 250 mL Erlenmeyer flasks, containing 90 mL of medium O, followed by growth for another 2–3 h (until OD_600nm_ = 1.0–1.2 was achieved).

### 2.4. Fermentation Assays

#### 2.4.1. Semi-Defined Fermentation Media

Simple semi-defined media, consisting of glucose (G), fructose (F), or galactose (Ga) at different concentrations (30, 50, 70, 90, 110, and 130 g/L), as well as mixture semi-defined media with a similar composition of sugars present in CD enzymatic hydrolysate (G + F, 40 + 20 g/L; and G + Ga, 40 + 20 g/L), were prepared and used for BDO production. These simple and mixture semi-defined media were chosen regarding the composition of the CD enzymatic hydrolysate. The mixture G + F + Ga was not tested, as the HPLC column used to measure sugars (Aminex HPX-87H column, see Section 2.5) is not able to separate the fructose and galactose sugars. Both pre-culture media H and O employed in the inoculum preparation were used as supplements in all semi-defined media. Nutrients and sugar solutions were sterilized at 121 °C for 15 min; while the potassium phosphate buffers (pH 6 and 6.5) and trace element solutions were sterilized by filtration (using 0.2 μm cellulose nitrate filters).

Fermentation assays were carried out, using a rotary shaker, in 250 mL Erlenmeyer flasks (containing 100 mL of medium) at 37 °C, 200 rpm, 144 h, and pH 6 (medium H) or 6.5 (medium O). The inoculum loading used was 10% (*v*/*v*), and no control of pH was employed during the fermentation. Samples were withdrawn each 24 h, centrifuged (at 13,500 rpm for 10 min), and their contents in sugars, BDO, ethanol, acetoin, and cells were measured. All fermentation tests were performed at least in duplicate.

#### 2.4.2. Carrot Discard (CD) Enzymatic Hydrolysate-Based Fermentation Medium

In order to be used as the fermentation medium for BDO production, the carrot discard enzymatic hydrolysate (CDEH) was supplemented with the same nutrients used in the pre-culture medium H, except glucose and yeast extract, pasteurized at 90 °C for 15 min and adjusted to pH 6 with KOH 10 M.

Fermentation tests were performed in a rotary shaker under the same conditions used for assays in semi-defined media, but employing, in this case, different stirring speeds (100, 200, and 300 rpm). Moreover, experiments at 200 rpm and different tryptone concentrations (0, 1, 2.5, and 5 g/L) were also carried out in order to evaluate the influence of the nitrogen source. Samples were also withdrawn each 24 h, centrifuged, and analyzed for their content in sugars, BDO, ethanol, acetoin, and cells. All fermentation tests were performed at least in duplicate.

In both semi-defined and CDEH media, the yields and productivities of BDO were calculated. The BDO yield (g BDO/g substrate (sugars) consumed) was calculated as the relation between the BDO concentration (g/L) achieved in fermentation tests and the concentration of substrate (sugars) (g/L) consumed during fermentation. On the other hand, BDO productivity (g/L·h) was calculated as the ratio between the BDO concentration (g/L) and the fermentation time (h) at which this BDO concentration was measured.

### 2.5. Analytical Methods

In order to analyze the content of the extractives, structural carbohydrates, lignin, and ash in CD, analytical methods from the National Renewable Energy Laboratory (NREL) [29,30,31] were used. High-performance liquid chromatography (HPLC) determined the content of galacturonic acid, sugars (glucose, galactose + fructose, and arabinose), and fermentation products (BDO, ethanol, and acetoin), using a refractive index detector (Waters 2414), an Aminex HPX-87H column (at 60 °C), and 0.01 N H_2_SO_4_ (0.6 mL/min) as the mobile phase. Cell concentration in the fermentation tests, which were determined by the dry weight method, filtering the samples through 0.2 μm cellulose nitrate filters (Sartorius 254 stedim Biotech, Göttingen, Germany), was calculated as the ratio between the dried mass of the biomass and the volume of the filtered sample.

All analytical determinations were carried out in triplicate and the average results are shown.

### 2.6. Data Analysis

To determine statistical differences, an ANOVA was carried out, at a confidence level of 95% (*p* < 0.05). A Tukey multiple range test was carried out using Statgraphics Centurion XVIII.

## 3. Results and Discussion

### 3.1. BDO Production from Semi-Defined Media: Influence of Pre-Culture and Substrate

In order to evaluate the influence of different types of substrate (contained in CDEH) and pre-culture media in *P. polymyxa*, diverse fermentation tests with semi-defined media were carried out. Experiments were firstly conducted using simple sugars, glucose (G), fructose (F), or galactose (Ga), at different concentrations (30, 50, 70, 90, 110, and 130 g/L). As was previously indicated, two growth media, H and O, were tested, in order to determine the optimum growth conditions for the microorganism. The main difference between both growth media is related to nitrogen source, since O medium includes tryptone in the composition. Once the best growth medium was evaluated, tolerance to mixed sugars was also studied.

A good cultivation strategy is necessary to enhance BDO production [9]. The maximum values for BDO concentration, yield, and productivity attained were also studied (Table 1).

#### 3.1.1. Tolerance of *P. polymyxa* to Simple Sugars

**Figure 1:**
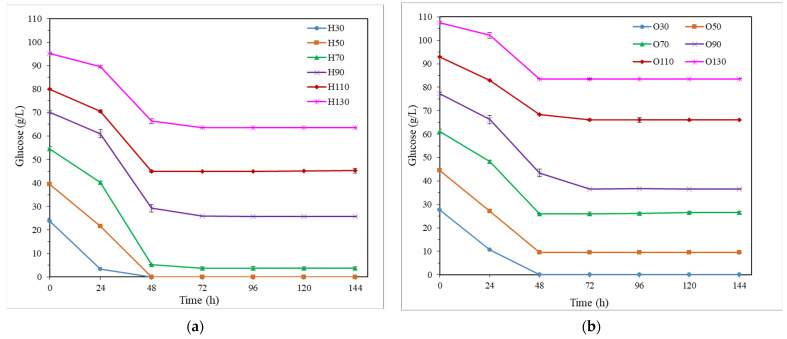

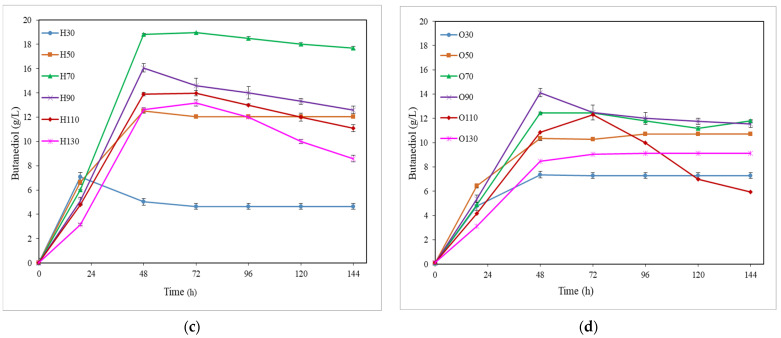
Fermentation kinetics in semi-defined media of glucose. Glucose consumption (**a**) and butanediol production (**b**) in Häßler medium (H) and glucose consumption (**c**) and 2,3-butanediol production (**d**) in Okonkwo medium (O).

Regarding the use of simple glucose (Figure 1 and Table 1), at the end of the fermentation process, the microorganism was able to assimilate completely the glucose contained in the media with 30 and 50 g/L glucose, and almost its totality (sugar uptake = 93.3%) for 70 g/L glucose, when the medium H was used (Figure 1a and Table 1). However, for glucose concentrations ≥ 90 g/L (Figure 1a), considerable glucose amounts (26–64 g/L glucose) were found at the end of the fermentation tests (sugar uptake = 33–63%). However, the medium O showed a lower glucose assimilation capacity (Figure 1c and Table 1), only achieving high sugar uptake when low glucose concentrations were employed (100 and 86% at the end of fermentation for 30 and 50 g/L glucose, respectively); with sugar uptake between 22% and 52% at the end of the fermentation process for higher glucose levels (70–130 g/L). By comparing the BDO production with both pre-culture media H and O (Figure 1b,d and Table 1), in general, higher maximum BDO concentrations (7.4–14.1 g/L) were obtained in all fermentation tests when the medium H was used. These maximum values were reached between 24 and 48 h of fermentation (except for the tests with the highest glucose concentrations, 110 and 130 g/L, when 72 h was necessary to attain the maximum BDO concentration). These results are similar to those reported in the literature, with maximum productions of BDO after 36–48 h when up to 100 g/L glucose is employed [1].

**Figure 2:**
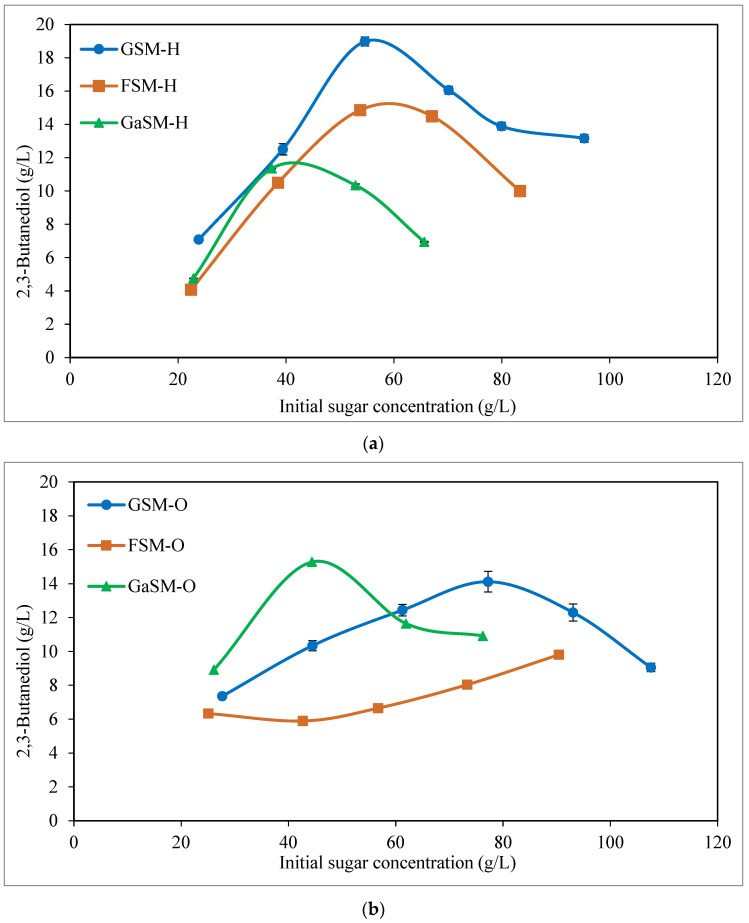
Maximum levels of 2,3-butanediol (g/L) obtained in the fermentation assays in semi-defined media of glucose, fructose, and galactose, using both Häßler (**a**) and Okonkwo (**b**) media. GSM-H: glucose semi-defined medium—Häßler medium; FSM-H: fructose semi-defined medium—Häßler medium; GaSM-H: galactose semi-defined medium—Häßler medium; GSM-O: glucose semi-defined medium—Okonkwo medium; FSM-O: fructose semi-defined medium—Okonkwo medium; GaSM-O: galactose semi-defined medium—Okonkwo medium. Maximum BDO concentration corresponds to fermentation time indicated in Table 1.

Figure 2 shows the maximum levels of BDO achieved vs. the initial sugar concentrations for each type of sugar and the concentrations used in the different fermentation tests. A maximum BDO value as high as 19.0 g/L was obtained when the medium H and an initial glucose concentration of 70 g/L were used; while BDO concentrations were lower than 14.1 g/L for the medium O. The initial sugar concentrations (t = 0) measured in the different fermentation assays (shown, for example, in Figure 2) are different from the real ones initially put into the substrate medium. This is because, during the preparation process of the substrate medium, different volumes of potassium phosphate buffer, trace elements, and inoculum were added to the initial prepared substrate medium; so the initial prepared sugar concentrations (semi-defined media) were diluted.

On the other hand, as can be seen in Table 1, in general, high BDO yields were obtained in all the fermentation tests (0.32–0.42 and 0.27–0.46 g/g for the media H and O, respectively) at the time of maximum butanediol production; the highest BDO yields being achieved, in general, for both media H and O when the highest initial glucose concentrations (110 and 130 g/L) were used. Therefore, taking into account the fact that the theoretical BDO yield from glucose, fructose, or galactose is considered to be 0.5 g/g [31], BDO yields of up to 92% of their theoretical value were reported in this work. BDO productivities ranged from 0.18 to 0.37 g/L·h and from 0.13 to 0.29 g/L·h for H and O media, respectively (Table 1).

Fermentation of glucose semi-defined media (135 g/L) by *Klebsiella neumoniae* with ammonium phosphate as nitrogen source led up to 52.4 g/L of BDO (yield of 0.45 g/g) [32,33]. With genetically modified *Escherichia coli*, up to 7.14 g/L BDO and a yield of 0.29 g/g were reported [34]. The use of *Serratia marcescens* LQOB-SE6 provided up to 30 g/L BDO after 5 days of cultivation [35] when 75 g/L of glucose was employed (yield of 0.42 g/g). Lee et al. [36] reported 31.5 g/L BDO from 80 g/L glucose in batch fermentation with genetically modified *Saccharomyces cerevisiae*. Okonkwo et al. [1] obtained up to 32.2 g/L BDO (yield of 0.33 g/g) from 100 g/L glucose and *P. polymyxa* and Schilling et al. [37] obtained 48.5 g/L BDO (yield of 0.43 g/g) from 140 g/L glucose and modified *P. polymyxa.*

**Figure 3:**
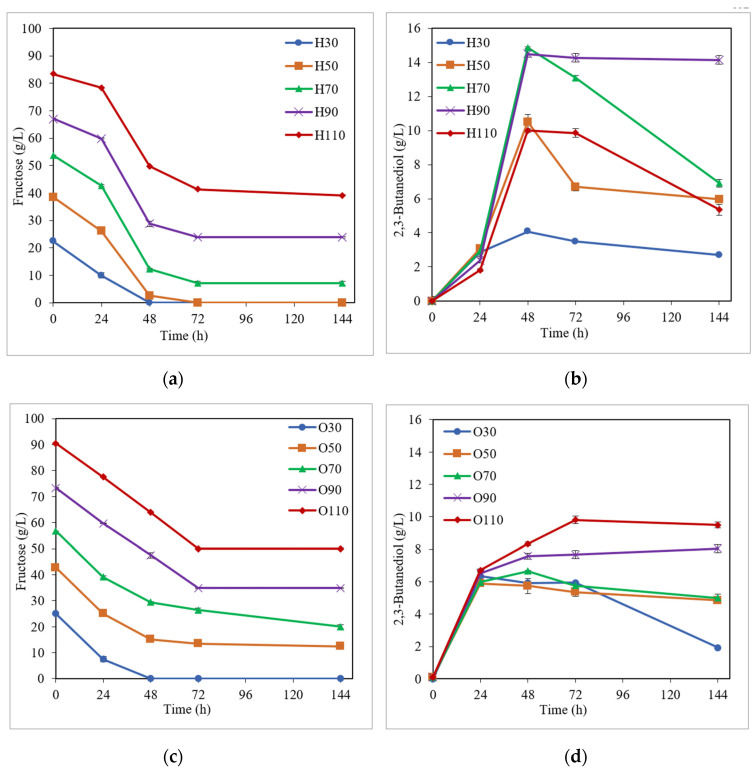
Fermentation kinetics in semi-defined media of fructose. Fructose consumption (**a**) and butanediol production (**b**) in Häßler medium (H) and fructose consumption (**c**) and 2,3-butanediol production (**d**) in Okonkwo medium (O).

**Figure 4:**
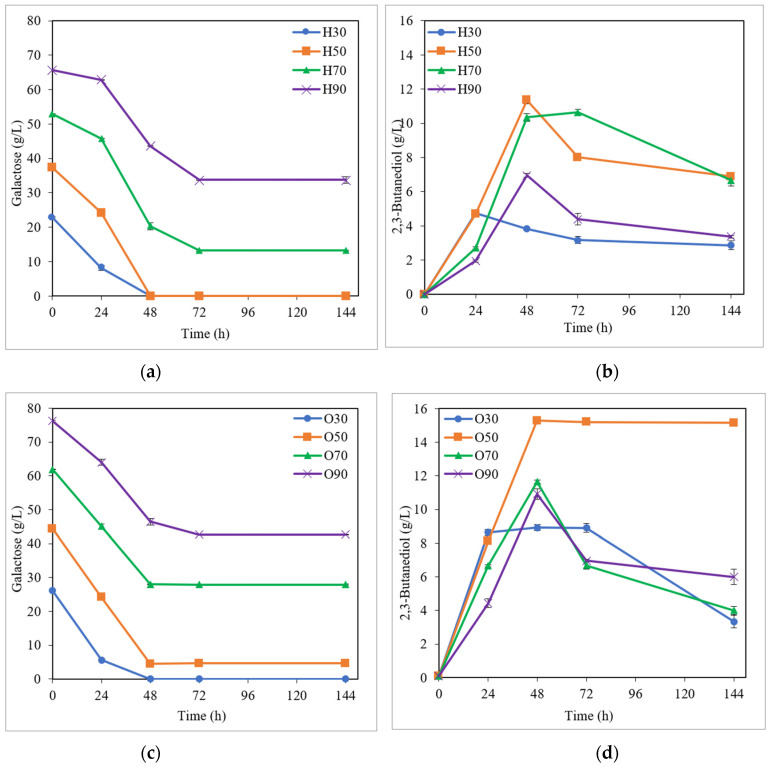
Fermentation kinetics in semi-defined media of galactose. Galactose consumption (**a**) and butanediol production (**b**) in Häßler medium (H) and galactose consumption (**c**) and 2,3-butanediol production (**d**) in Okonkwo medium (O).

Concerning the substrate media based on simple fructose and galactose (Figure 3 and Figure 4 and Table 1), a similar behavior was observed in the sugar consumption to that described before for the glucose media. As can be appreciated in Figure 3a and Figure 4a, and Table 1, a high sugar uptake (86.9–100.0% and 75.0–100.0% for media based on fructose and galactose, respectively) was achieved at the end of fermentation for initial sugar concentrations between 30 and 70 g/L when the medium H was used; while considerable sugar consumption (70.9–100.0% and 90.0–100.0% for media based on fructose and galactose, respectively) was noted for 30 and 50 g/L initial sugar using the medium O (Figure 3c and Figure 4c, and Table 1). For higher fructose and galactose levels (≥90 g/L), as can be seen in Table 1, the sugar uptake decreased in both H and O media. With regard to the BDO production (Figure 3b,d and Figure 4b,d, and Table 1), for semi-defined media based on fructose, the highest BDO concentrations (ranging from 4.1 to 14.9 g/L) were attained for the medium H (except for 30 g/L of initial fructose) (Figure 3b,d and Table 1), similar to that observed using glucose in the substrate medium; while, when simple galactose was used as the substrate, the highest BDO levels (8.9–15.3 g/L BDO) were obtained for the medium O (Figure 4b,d and Table 1). This same behavior can also be observed in Figure 2, with the highest BDO concentrations being obtained at 70 g/L initial fructose (14.9 g/L BDO) for medium H and at 110 g/L initial fructose (9.8 g/L BDO) for medium O; while, when using galactose as the substrate medium, the highest BDO levels were yielded at 50 g/L initial galactose for both H and O media (11.4 and 15.3 g/L BDO, respectively). Furthermore, as can be seen in Table 1, high BDO yields and productivities were also attained in fermentation tests with simple fructose and galactose, reaching values of up to 0.38 g/g (76% of its theoretical value) and 0.31 g/L·h for both fructose and galactose semi-defined media. Cell concentrations (at the time of maximum BDO production) were not very different for the two pre-culture media, using both fructose (1.9–3.3 and 0.8–3.9 g/L for H and O media, respectively) and galactose (1.5–2.7 and 1.6–2.5 g/L for H and O media, respectively) semi-defined media (Table 1).

A few studies were found in the literature concerning the use of semi-defined media with other simple sugars than glucose. The literature reported the use of sucrose providing 8.62 g/L of BDO (yield of 0.30 g/g) when modified *Vibrio natriegens* was employed as the microorganism [34] and the use of glucose or pentoses (xylose and arabinose), showing a preference order of glucose > xylose > arabinose [1].

In summary, the results indicate that the order of preference of *P. polymyxa* was glucose > fructose > galactose when H growth medium was employed in the range of 40–80 g/L of initial sugars, whereas galactose was preferred when O growth medium was used.

#### 3.1.2. Influence of Mixed Sugars in 2,3-Butanediol Production

**Figure 5:**
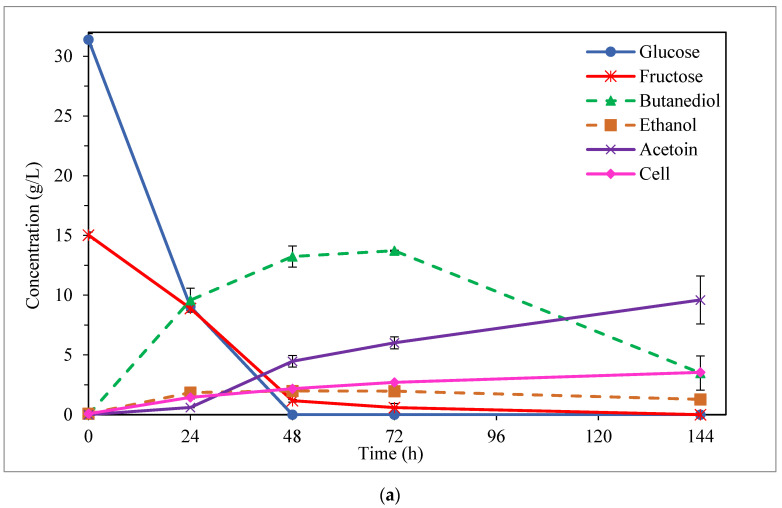

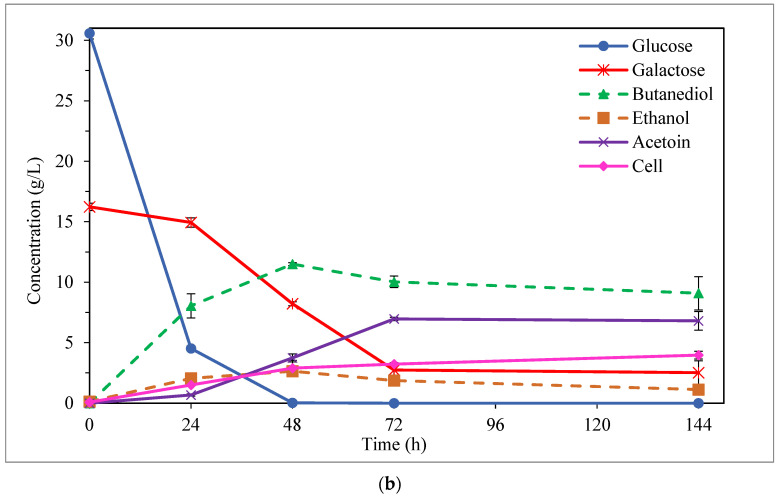
Fermentation kinetics in semi-defined media of sugar mixtures: glucose + fructose (**a**) and glucose + galactose (**b**), using Häßler medium.

Furthermore, a mixture semi-defined medium with a similar composition of sugars to that contained in CD enzymatic hydrolysate (G + F, 40 + 20 g/L; G + Ga, 40 + 20 g/L) was tested. In this case, only the pre-culture medium H was employed as this was considered, in general, to be the most adequate pre-culture medium when semi-defined media based on simple glucose and fructose were used (as these are the main sugars found in CDEH), as described above in this section. Figure 5 shows both the consumption of sugar (glucose, fructose, and/or galactose) and the BDO production for both mixtures of semi-defined media: G + F (Figure 5a) and G + Ga (Figure 5b). As can be observed, in both mixtures of the semi-defined media, the glucose was totally consumed at 48 h of fermentation, while a high uptake of this sugar can already be appreciated at 24 h of the process (71.2 and 85.2% for G + F and G + Ga media, respectively). However, although most of the fructose (92.2%) had already been assimilated by *P. polymyxa* at 48 h of fermentation in the G + F medium (being consumed completely at the end of the process) (Figure 5a), the highest galactose consumption was reached after 72 h in the G + Ga medium (83.1%) (Figure 5b). Whereas glucose and fructose were co-utilized to a reasonable degree, galactose was co-used to a low degree until most of the glucose was assimilated (24 h), glucose then being the preferred substrate (Figure 5). In this case, the glucose utilization (24 h) was 10.7 times greater than that of galactose. This behavior was also observed by Okonkwo et al. [1] in the fermentation of mixtures of semi-defined media, but when mixing glucose with pentoses (xylose and arabinose). Regarding the BDO production, as can be seen in Figure 5 and Table 1, the maximum BDO concentrations of up to 13.7 and 11.5 g/L were attained for the G + F and G + Ga media, respectively (at 72 and 48 h of fermentation, respectively). The highest BDO levels were achieved for the G + F mixture (an increase of 16.1% compared to G + Ga). On the other hand, even though the BDO productivity (at the time of maximum butanediol production) was higher for the G + Ga medium (0.24 vs. 0.19 g/L·h), similar BDO yields (0.30 g/g, 60% of their theoretical value) were obtained for both mixture media (Table 1); these values being of the same order as those achieved using simple sugar media with similar concentrations to those found in these mixture media (40 g/L glucose and 20 g/L fructose or galactose). Cell concentrations (at the time of maximum BDO production) were also very similar for both mixture media (2.7 and 2.9 g/L for the G + F and G + Ga media, respectively) (Table 1).

Therefore, in conclusion, the pre-culture medium H was considered, in general, to be the most adequate when semi-defined media were employed; so, it will be used in subsequent fermentation tests of carrot discard.

### 3.2. BDO Production from Enzymatic Hydrolysate of Carrot Discard

**Figure 6:**
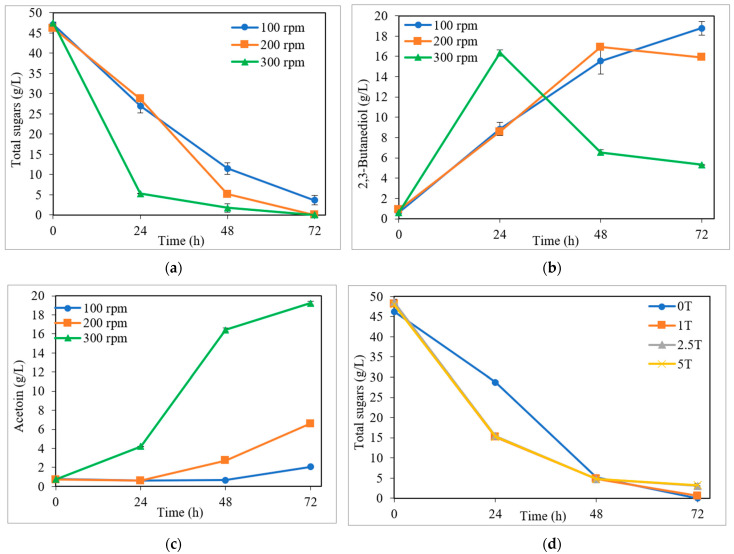

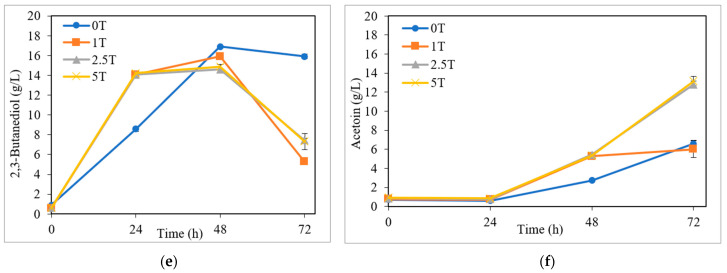
Fermentation kinetics in enzymatic hydrolysate of carrot discard, using the Häßler medium. Total sugar consumption (**a**,**d**) and 2,3-butanediol (**b**,**e**) and acetoin (**c**,**f**) production for different stirring speeds (100, 200, and 300 rpm) (**a**–**c**) and tryptone concentrations, T (0, 1, 2.5, and 5 g/L) (**d**–**f**).

Carrot discard enzymatic hydrolysate (CDEH) was used as the fermentation medium for BDO production by *P. polymyxa* under the same fermentation conditions used for semi-defined media (200 rpm and 0 g/L tryptone). The total sugar content of CDEH was 56.5 g/L, its composition being the following (g/L): glucose, 34.3; fructose + galactose, 20.3; arabinose, 1.9; acetic acid, 0.6; and total phenols, 0.8. As indicated before for semi-defined media, the initial sugar concentrations (t = 0) measured in the CDEH fermentation assays (Figure 6a,d) were slightly different from the real one indicated before for CDEH (56.5 g/L). This is because, during the preparation process of the fermentation assays, different volumes of potassium phosphate buffer, trace elements, and inoculum were added to the CDEH fermentation medium; so, the initial sugar concentration of the CDEH was diluted. The pre-culture medium H was used, as this was considered, in general, to be the most adequate pre-culture medium when semi-defined media were used, as described in Section 3.1.1.

Compared to the mixtures of semi-defined media (G + F and G + Ga) (Section 3.1.1.), as can be seen in Figure 6a,d and Table 2, at 200 rpm and without tryptone, the totality of sugars contained in the CDEH were assimilated by *P. polymyxa* at 72 h of fermentation, 89% of the total sugars already being consumed at 48 h of the process. Considering the simple sugars (glucose, fructose + galactose (FGa), and arabinose), all sugars were simultaneously consumed during the fermentation process, the glucose being consumed in its totality at 48 h, with 75.9% and 28.1% of FGa and arabinose, respectively, also having been consumed at this fermentation time. This behavior is similar to that found with mixtures of the semi-defined media (G + F and G + Ga).

Regarding BDO production, as can be appreciated in Figure 6b,e, a maximum concentration of 16.9 g/L was achieved at 48 h of fermentation (at 200 rpm and without the presence of tryptone to compare with the results obtained using mixtures of semi-defined media), resulting in a BDO yield and productivity of 0.41 g/g (82% of its theoretical value) and 0.35 g/L·h, respectively (Table 2). So, the results obtained from the CDEH fermentation medium are much better than those attained for mixtures of semi-defined media (G + F and G + Ga, Table 1) (0.41 vs. 0.30 g/g and 0.35 vs. 0.19–0.24 g/L·h). This is probably due to the presence of different compounds (such as proteins, carotenes, calcium, and phosphorous) in carrot discard [26] that could be beneficial for *P. polymyxa*.

On the other hand, in order to try to enhance these results, the influences of stirring/aeration and the presence of tryptone were also analyzed using CDEH as the fermentation medium.

#### 3.2.1. Influence of Stirring

One of the most important parameters in BDO fermentation is stirring, which could be used as a simple oxygen supply method [5]. In order to produce BDO efficiently, the determination of the optimum stirring speed is crucial, as it also greatly depends on the microorganism used in the fermentation process [9]. In this way, the influence of stirring in BDO fermentation by *P. polymyxa* was evaluated, using CDEH as the fermentation medium.

Figure 6a,b and Table 2 show the total sugar uptake and the BDO production obtained when different stirring speeds (100, 200, and 300 rpm) were employed in the fermentation tests. As can be seen, *P. polymyxa* was able to consume high percentages of the total sugars in all cases, with the totality of sugars contained in CDEH being assimilated at 72 h fermentation when stirring speeds of 200 and 300 rpm were used and, at this process time, 7.8% of the initial total sugars remained unconsumed at 100 rpm. The rate of sugar consumption increased for higher stirring speeds, with 90% of the total sugars being consumed at 24 h of fermentation at 300 rpm, 89% at 48 h at 200 rpm, and 92% at 72 h of fermentation at 100 rpm (Figure 6a). Cho et al. [38] also observed that the increase in stirring speeds led to a better glucose assimilation by *Klebsiella oxytoca* M1. As described above for CDEH fermentation at 200 rpm (Section 3.2), the simple sugars (glucose, FGa, and arabinose) were simultaneously consumed by *P. polymyxa* during the fermentation process, showing a higher preference for glucose as compared to FGa and arabinose.

On the other hand, as can be observed in Figure 6b and Table 2, the highest maximum BDO concentration (18.8 g/L) was achieved when the lowest stirring speed (100 rpm) was used; while longer fermentation times were necessary (72 h for 100 rpm vs. 48 and 24 h for 200 and 300 rpm, respectively). This same behavior was also observed for the BDO yield, with the highest value being obtained for the lowest stirring speed (100 rpm) (0.43 g/g (86% of its theoretical value) vs. 0.41 and 0.39 g/g for 200 and 300 rpm, respectively) (Table 2). This change could be due to a high accumulation of ethanol and acetoin, which are the main by-products in the BDO fermentation process [9,39], as described in the next section. In this context, as can be seen in Figure 6, when 200 and 300 rpm were used, *P. polymyxa* continued to consume sugars once the maximum BDO production (at 48 and 24 h for 200 and 300 rpm, respectively) had been reached, which were used for acetoin and cell production (Section 3.3). Nevertheless, a contrary trend was appreciated for BDO productivity, with this parameter being increased along with the stirring speed, reaching the highest value (0.68 g/L·h) for 300 rpm (Table 2). Park et al. [40] reported this same behavior in BDO fermentation tests by *K. oxytoca*, where BDO productivity improved from 0.43 to 2.7 g/L·h when the stirring speed increased from 150 to 450 rpm. Xu et al. [41] also showed a great enhancement in BDO productivity from 0.51 g/L·h to 1.48 g/L·h when the stirring speed increased from 200 rpm to 400 rpm.

Therefore, the use of a fixed stirring speed during the whole fermentation process would not be adequate for the production of high BDO concentrations, yields, and productivities; a stirring speed control in two steps thus being a strategy of interest. In this way, for instance, the use of a high agitation speed (i.e., 300 rpm) during the first hours of fermentation (i.e., about 15 h) would allow a high cell growth to be obtained, while the subsequent the use of a lower agitation speed (i.e., 200 rpm) would allow an increase in the 2,3-butanediol accumulation. This would therefore allow enough oxygen supply while also enhancing the BDO production [42,43].

#### 3.2.2. Influence of the Presence of Tryptone

Another of the most influential factors in BDO production is the presence of tryptone (T), which is a very important nutrient (organic nitrogen source) for cell growth [28]. The influence of the presence of tryptone in different concentrations (0, 1, 2.5, and 5 g/L) as a supplement in the CDEH fermentation medium was analyzed in the BDO fermentation assays by *P. polymyxa*.

Figure 6d,e and Table 2 show the total sugar consumption and BDO production obtained when different tryptone concentrations (0, 1, 2.5, and 5 g/L) were employed. As can be appreciated in Figure 6d, solely for low tryptone levels (0 and 1 g/L), the totality of sugars assimilated by *P. polymyxa* at the end of the fermentation process (72 h) was 6.3% and 6.7% of the total unconsumed sugars remaining when the CDEH was supplemented with 2.5 and 5 g/L tryptone, respectively. However, the presence of tryptone, independently of the concentration used, led to higher rates of sugar consumption for the first hours of fermentation, with a sugar uptake of 68% when tryptone was used, versus 37.6% without the presence of tryptone at 24 h of the process (Figure 6d). In this case, the simple sugars (glucose, FGa, and arabinose) were also simultaneously consumed by *P. polymyxa* during the fermentation process, showing a higher preference for glucose as compared to FGa and arabinose.

On the other hand, as can be observed in Figure 6e and Table 2, the maximum BDO production (at 48 h of the process in all cases) decreased when higher tryptone concentrations were used, with no influence of tryptone for T > 2.5 g/L. Therefore, the highest values of BDO concentration, yield, and productivity (16.9 g/L, 0.41 g/g, and 0.35 g/L·h, respectively) were attained when the CDEH was fermented with no tryptone supplementation, thus yielding 82% of its theoretical value (Table 2).

However, although the presence of tryptone did not enhance BDO production, the cell concentration increased with the use of higher tryptone concentrations (Table 2). This same behavior was also observed by Okonkwo et al. [28], who evaluated the impact of the presence of tryptone (ranging between 5 and 7 g/L) in BDO fermentation by *P. polymyxa* DSM 365, using the response surface methodology. They concluded that tryptone had a negative effect on BDO production but a positive effect on cellular growth. This could be due to tryptone acting as a source of amino acids for protein biosynthesis (such as enzymes) and of nitrogen for nucleic acid biosynthesis [28].

In short, the best results obtained in this work from CDEH (18.8 g/L BDO and 18.8 g BDO/100 g carrot discard), with a BDO yield and productivity of 0.43 g/g (86% of its theoretical value) and 0.26 g/L·h, respectively, were achieved at 100 rpm, with no tryptone, the pre-culture medium H, and the use of pre-treatment for carrot discard not being necessary, unlike for other fruit and vegetable residues such as apple pomace [44], where a hydrothermal pre-treatment prior to the enzymatic hydrolysis process was required for BDO production. Comparing the results obtained in this study with those reported in the literature for other fruit and vegetable residues, for instance, a similar BDO concentration (18.2 g/L) and a lower BDO yield (0.36 vs. 0.43 g/g) were reported by Liakou et al. [19] in the BDO fermentation by *Enterobacter ludwigii* FMCC 204 (also using a shake flask as in this work) from fruit waste (plums, apples, and pears) extract (with 50 g/L initial total sugar concentration, not very different to that used in this work), which was obtained through sequential maceration, suspension in water, and centrifugation. Although a similar BDO yield (0.43 g/g) to that achieved in this work was obtained by *Klebsiella pneumoniae* PM2 from the whole slurry of oil palm empty fruit bunches (EFBs) generated by sulfite pre-treatment (1:4 (*w*/*v*) solid/liquid ratio, 165 °C, 75 min, and 7% (*w*/*w*) sodium bisulfite and 2.5% (*w*/*w*) sulfuric acid) [45], only 13.5 g BDO/100 g EFB (vs. 18.8 g BDO/100 g CD achieved in this work) was reported in this case. BDO was also generated by *K. pneumoniae* PM2 from EFB enzymatic hydrolysate, which was previously subjected to a two-stage organosolv pre-treatment (first stage: 1:4 (*w*/*v*) solid/liquid ratio, 170 °C, 40 min and 65% 1,4-BDO (*w*/*w*); second stage: 1:3 (*w*/*v*) solid/liquid ratio, 170 °C, 20 min, and 15 mM H_2_SO_4_), resulting in a slightly higher BDO yield than that obtained in this work (0.45 vs. 0.43 g/g) [46]. However, much lower BDO concentrations and yields (12.80 g/L and 0.17 g/g, respectively) than those achieved in this work (18.8 g/L and 0.43 g/g, respectively) were attained by Białkowska et al. [44], using *Bacillus subtilis* LOCK 1086, from apple pomace hydrolysate (with 40 g/L initial total sugar concentration), which was obtained through sequential hydrothermal pre-treatment (at 121 °C for 20 min) and enzymatic hydrolysis (by *A. niger* IBT 90). OHair et al. [47] also reported much lower BDO concentrations (5.2–5.9 g/L) and slightly lower BDO yields (0.38–0.41 g/g) than those achieved in this work in the fermentation of aqueous solutions of pepper, pineapple, and cabbage waste by *Bacillus licheniformis* YNP5-TSU.

### 3.3. By-Product Formation: Ethanol and Acetoin

Ethanol and acetoin, which are two of the main by-products from BDO fermentation [9], were found as by-products in the fermentation assays in both semi-defined media and CDEH. Both by-products come from pyruvate, a key intermediate in the BDO fermentation process, with ethanol being generated through successive pyruvate–formate lyase, acetaldehyde dehydrogenase, and ethanol dehydrogenase pathways; while acetoin is produced by successive α-acetolactate synthase and 4,α-acetolactate decarboxylase pathways [9].

Ethanol is one of the most interesting biofuels that can be obtained from renewable biomass, being a realistic short-term replacement for fossil fuels [48]. As can be seen in Table 1, using the H medium, ethanol concentrations (at the time of maximum butanediol production) ranged from 0.9 to 2.4, 1.9 to 4.3, and 1.3 to 2.6 g/L for semi-defined media of simple glucose, fructose, and galactose, respectively. However, when the O medium was used, ethanol levels were, in general, lower in all fermentation assays carried out (0.4–1.2, 0.6–1.9, and 0.8–2.2 g/L for semi-defined media of simple glucose, fructose, and galactose, respectively). Very similar ethanol amounts were also achieved using a mixture of semi-defined media (2.0 and 2.6 g/L for G + F and G + Ga media, respectively) (Table 1) and CDEH (1.8 g/L, under the same fermentation conditions used for semi-defined media: 200 rpm and without tryptone) (Table 2) as fermentation media.

When the stirring speed and presence of tryptone were studied in the fermentation process using CDEH as the fermentation medium, as can be appreciated in Table 2, the increase in the stirring speed (from 100 to 300 rpm) and the presence of tryptone (at 1, 2.5 and 5 g/L) led to a decrease in ethanol production (from 2.5 to 1.5 and 1.8 to 1.2 g/L, respectively). This behavior was similar to that observed for BDO production (Section 3.2.1 and Section 3.2.2 and Table 2). The presence of ethanol was low in all cases, which is desirable, since the generation of this by-product negatively influences the BDO fermentation yields [9].

Regarding acetoin production, as can be observed in Table 1, acetoin concentrations (at the time of maximum butanediol production) ranging from 1.3 to 3.5, 0.6–8.6, and 1.3–2.7 g/L were found for semi-defined media of simple glucose, fructose, and galactose, respectively. Interestingly, in this case, the acetoin levels found were, in general, higher for the O medium compared to those detected with the H medium, unlike those attained for the ethanol by-product. Relatively appreciable acetoin values were also detected when the mixture of semi-defined media was used (3.7 and 6 g/L for G + Ga and G + F, respectively) (Table 1).

On the other hand, as can be seen in Table 2, using CDEH as the fermentation medium, the increase in the stirring speed (from 100 to 300 rpm) and the presence of tryptone (at 1, 2.5, and 5 g/L) resulted in a higher acetoin production (from 2.1 to 4.2 and 2.7 to 5.4 g/L, respectively, at the time of maximum butanediol production). Furthermore, as can be observed in Figure 6c,f, at the end of the fermentation process (72 h), acetoin values of up to 9.3 times greater (at 300 rpm) were detected as compared to using 100 rpm (Figure 6c); while the use of 5 g/L tryptone resulted in acetoin values two times greater than without tryptone (Figure 6f). In both cases (Figure 6c,f), the considerable acetoin production started when low sugar concentrations remained without being consumed in the fermentation medium, with the BDO generated also being considerably consumed (Figure 6). This behavior was also observed by Okonkwo et al. [1] in the BDO fermentation by *P. polymyxa* DSM 365 of non-detoxified wheat straw hydrolysates. The biological synthesis of BDO took place through the 2,3-BDO dehydrogenase metabolic pathway, and it was necessary to have NADH to reduce the acetoin to BDO [49]. However, according to Maina et al. [5], BDO can be reversibly turned into acetoin, regenerating the NADH and then keeping a continual oxidation–reduction state.

## 4. Conclusions

Carrot discard enzymatic hydrolysate (CDEH) can be used as the fermentation medium for BDO production by *Paenibacillus polymyxa* DSM 365. Pre-culture Häßler medium, a stirring of 100 rpm, and no tryptone (nitrogen source) were shown to be the best fermentation variables studied for BDO production. Yields of 18.8 g of BDO, as well as 2.5 g of ethanol and 2.1 g of acetoin by-products, per 100 g of carrot discard were attained from CDEH. No hydrothermal pre-treatment is required to obtain promising BDO production results, which is advantageous to ensure the profitability of the process. Therefore, this study demonstrates new opportunities for carrot discard valorization.

## Figures and Tables

**Table 1:** Fermentation assays in semi-defined media of glucose (G), fructose (F), and/or galactose (Ga), using Häßler (H) and Okonkwo (O) media. Sugar uptake (%), 2,3-butanediol (BDO), ethanol, acetoin, and cell concentrations (g/L), as well as butanediol yield (YBDO/sugars, expressed as g/g sugars consumed) and productivity (PBDO, expressed as g/L·h) at the time of maximum butanediol production, are all indicated in the table. Data in parentheses refer to sugar uptake at the end of the fermentation process (144 h).

Initial Sugar Conc. (g/L)	Fermentation Medium	Time (h)	Sugar Uptake (%)	BDO (g/L)	Ethanol (g/L)	Acetoin (g/L)	Cell (g/L)	Y_BDO/sugars_ (g/g)	P_BDO_ (g/L·h)
30 G	H	24	86.0 (100)	7.1 ± 0.3	0.9 ± 0.0	1.6 ± 0.1	1.6 ± 0.1	0.35	0.37
	O	48	100 (100)	7.4 ± 0.4	1.0 ± 0.0	3.5 ± 0.1	2.7 ± 0.0	0.27	0.15
50 G	H	48	100 (100)	12.5 ± 0.2	2.1 ± 0.1	2.8 ± 0.2	3.7 ± 0.3	0.32	0.26
	O	48	78.7 (86.0)	10.3 ± 0.3	1.2 ± 0.1	3.5 ± 0.6	2.8 ± 0.5	0.30	0.22
70 G	H	72	93.3 (93.3)	19.0 ± 0.0	2.4 ± 0.0	1.9 ± 0.2	3.4 ± 0.4	0.37	0.26
	O	48	57.5 (57.5)	12.4 ± 0.3	0.7 ± 0.0	2.5 ± 0.0	3.2 ± 0.1	0.35	0.26
90 G	H	48	58.3 (63.1)	16.1 ± 0.3	2.2 ± 0.1	1.6 ± 0.3	2.9 ± 0.4	0.39	0.34
	O	48	43.8 (52.7)	14.1 ± 0.0	0.7 ± 0.1	1.8 ± 0.5	3.1 ± 0.3	0.42	0.29
110 G	H	48	43.7 (43.7)	13.9 ± 0.1	2.2 ± 0.0	1.3 ± 0.1	1.6 ± 0.2	0.40	0.29
	O	72	29.0 (29.0)	12.3 ± 0.2	0.7 ± 0.1	2.0 ± 0.2	2.4 ± 0.3	0.46	0.17
130 G	H	72	33.2 (33.2)	13.2 ± 0.2	1.3 ± 0.1	1.3 ± 0.0	4.3 ± 0.3	0.42	0.18
	O	72	22.3 (22.3)	9.1 ± 0.1	0.4 ± 0.0	2.5 ± 0.1	2.8 ± 0.2	0.38	0.13
30 F	H	48	100 (100)	4.1 ± 0.1	1.9 ± 0.0	3.4 ± 0.1	1.9 ± 0.1	0.18	0.09
	O	24	70.1 (100)	6.3 ± 0.1	1.4 ± 0.0	1.8 ± 0.1	1.2 ± 0.0	0.36	0.26
50 F	H	48	93.2 (100)	10.5 ± 0.2	4.0 ± 0.1	2.2 ± 0.2	3.3 ± 0.2	0.29	0.22
	O	24	41.3 (70.9)	5.9 ± 0.1	1.0 ± 0.0	1.8 ± 0.1	0.8 ± 0.0	0.33	0.25
70 F	H	48	77.2 (86.9)	14.9 ± 0.4	4.3 ± 0.2	0.6 ± 0.0	2.2 ± 0.1	0.36	0.31
	O	48	48.1 (64.8)	6.7 ± 0.1	1.9 ± 0.1	4.5 ± 0.3	2.3 ± 0.1	0.24	0.14
90 F	H	48	57.1 (64.3)	14.5 ± 0.1	3.6 ± 0.1	0.6 ± 0.1	2.5 ± 0.3	0.38	0.30
	O	144	52.5 (52.5)	8.0 ± 0.3	0.6 ± 0.0	8.1 ± 0.4	3.9 ± 0.2	0.21	0.06
110 F	H	48	40.3 (53.1)	10.0 ± 0.0	3.1 ± 0.1	1.0 ± 0.1	1.9 ± 0.1	0.30	0.21
	O	72	44.8 (44.8)	9.8 ± 0.2	1.0 ± 0.1	8.6 ± 0.3	2.5 ± 0.3	0.24	0.14
30 Ga	H	24	64.2 (100)	4.8 ± 0.2	1.3 ± 0.2	1.4 ± 0.2	1.5 ± 0.1	0.32	0.20
	O	48	100 (100)	8.9 ± 0.2	0.8 ± 0.0	2.6 ± 0.2	1.7 ± 0.0	0.34	0.19
50 Ga	H	48	100 (100)	11.4 ± 0.4	2.6 ± 0.2	1.9 ± 0.1	2.7 ± 0.1	0.30	0.24
	O	48	90.0 (90.0)	15.3 ± 0.4	0.8 ± 0.1	1.9 ± 0.0	2.0 ± 0.3	0.38	0.32
70 Ga	H	48	61.6 (75.0)	10.4 ± 0.2	2.5 ± 0.3	1.3 ± 0.0	2.5 ± 0.2	0.32	0.22
	O	48	54.8 (54.8)	11.7 ± 0.3	2.2 ± 0.2	2.7 ± 0.2	1.6 ± 0.1	0.34	0.24
90 Ga	H	48	33.6 (48.6)	7.0 ± 0.1	1.4 ± 0.0	1.3 ± 0.1	1.5 ± 0.1	0.31	0.15
	O	48	39.1 (44.1)	10.9 ± 0.2	1.1 ± 0.1	1.4 ± 0.1	2.5 ± 0.3	0.37	0.23
G + F (40 + 20)	H	72	98.7 (100)	13.7 ± 0.1	2.0 ± 0.1	6.0 ± 0.5	2.7 ± 0.1	0.30	0.19
G + Ga (40 + 20)	H	48	82.5 (94.6)	11.5 ± 0.1	2.6 ± 0.1	3.7 ± 0.3	2.9 ± 0.3	0.30	0.24

**Table 2:** Fermentation assays in enzymatic hydrolysate of carrot discard, using Häßler medium and different stirring speeds (100, 200, and 300 rpm) and tryptone concentrations, T (0, 1, 2.5, and 5 g/L). Sugar uptake (%), 2,3-butanediol (BDO), ethanol, acetoin, and cell concentrations (g/L), and butanediol yield (Y_BDO/sugars_, expressed as g/g sugars consumed) and productivity (P_BDO_, expressed as g/L·h) at the time of maximum butanediol production are all indicated in the table. Data in parentheses refer to sugar uptake at the end of the fermentation process (72 h).

Initial Sugar Conc. (g/L)	Fermentation Medium	Time (h)	Sugar Uptake (%)	BDO (g/L)	Ethanol (g/L)	Acetoin (g/L)	Cell (g/L)	Y_BDO/sugars_ (g/g)	P_BDO_ (g/L·h)
Study of Stirring/Aeration									
100 rpm	H	72	92.2 (92.2)	18.8 ± 0.7	2.5 ± 0.1	2.1 ± 0.0	2.0 ± 0.3	0.43	0.26
200 rpm	H	48	88.9 (100)	16.9 ± 0.0	1.8 ± 0.0	2.7 ± 0.2	2.3 ± 0.1	0.41	0.35
300 rpm	H	24	89.0 (100)	16.3 ± 0.3	1.5 ± 0.2	4.2 ± 0.0	0.7 ± 0.2	0.39	0.68
Study of Tryptone Use in Fermentation Medium									
0 T	H	48	88.9 (100)	16.9 ± 0.0	1.8 ± 0.0	2.7 ± 0.2	2.3 ± 0.1	0.41	0.35
1 T	H	48	90.0 (98.8)	15.9 ± 0.1	1.3 ± 0.0	5.3 ± 0.3	2.5 ± 0.6	0.37	0.33
2.5 T	H	48	90.2 (93.7)	14.6 ± 0.0	1.3 ± 0.0	5.4 ± 0.0	3.0 ± 0.6	0.33	0.30
5 T	H	48	90.1 (93.2)	14.9 ± 0.2	1.2 ± 0.1	5.3 ± 0.0	3.0 ± 0.2	0.34	0.31

## Data Availability

Data will be available upon request.
